# Prevalence of faecal incontinence and its related factors among patients in a Malaysian academic setting

**DOI:** 10.1186/1471-230X-14-95

**Published:** 2014-05-18

**Authors:** April C Roslani, Rajeshwary Ramakrishnan, Soraya Azmi, Daryl J Arapoc, Adrian Goh

**Affiliations:** 1 Department of Surgery, University of Malaya Medical Centre, Kuala Lumpur, Malaysia; 2 Azmi Burhani Consulting Sdn Bhd, Petaling Jaya, Selangor, Malaysia; 3 Veras Research Sdn Bhd, Petaling Jaya, Selangor, Malaysia

**Keywords:** Faecal incontinence, Prevalence, Sphincter defects, Soiling, Constipation

## Abstract

**Background:**

Prevalence data is essential for planning of healthcare services. The prevalence of faecal incontinence (FI) varies worldwide, and in Malaysia is not known. We sought to estimate its prevalence among patients with various conditions in a Malaysian academic setting.

**Method:**

A questionnaire-based survey was conducted among a convenience sample of adult patients and relatives who visited the Obstetrics and Gynaecology and General Surgery Clinics of University of Malaya Medical Centre (UMMC) from June 2009 to February 2010. Data collected included patient demographics and pre-existing medical conditions known to be FI risk factors. Severity of FI was assessed using the Wexner Continence Scale (WCS).

**Results:**

Among the 1000 subjects recruited into the study, 760 (76%) were female and the median age was 38 years with an inter-quartile range of 24 years. The prevalence of FI among the study subjects was found to be 8.3%. Among them, 63 subjects (75.9%) were determined to have mild FI as measured by the WCS. The proportions of patients with moderate and severe FI were 18.3% and 6.0%, respectively. FI was found to be significantly associated with older age, presence of diabetes mellitus and increased duration of defaecation. There was no statistically significant association between FI and sex, defaecation frequency, or history of surgery.

**Conclusion:**

FI in our setting is prevalent enough to warrant targeted healthcare interventions, including the need to improve general public awareness of the condition in order to counter social stigma and embarrassment that may be faced by patients.

## Background

Faecal incontinence (FI) is defined as inappropriate or involuntary loss of flatus, liquid and stool with up to half of patients having rectal hypersensitivity and increased stool frequency and urgency [1]. This condition is associated with aging and loss-of-function or damage of anal sphincters as a result of childbirth and anal surgery [2,3]. FI interferes with many activities of daily living including sleep, work and social activities. It can be distressing, embarrassing and lead to social isolation, low self-esteem, reduced intimacy and also anxiety and depression [4,5]. FI has been referred to as the ‘silent affliction’ of ‘unvoiced symptoms’ [6]. Owing to the reluctance of patients to come forward, its reported prevalence tends to be underestimated and even unrecognized in many cases. Even though FI is estimated from previous studies to affect about 10% of the community, only a small minority seek medical attention [7,8]. Despite the anticipated difficulties of identifying patients for a study, we felt that attempting to understand the occurrence of the condition is important to better help patients who are faced with this socially challenging condition.

Earlier population based studies have tended to focus on elderly patients [9,10]. More recent studies have highlighted the problem of FI among women [11,12], although it should be noted that FI is a significant health issue among men as well [2,13]. Few studies have been conducted on the prevalence of FI among Asian populations, and the available studies have reported varying FI prevalence rates. A 1997 study, among a randomly selected sample of Japanese patients aged above 64 years, reported FI prevalence of 8.7% among males and 6.6% among females respectively [14] while a 2003 study of 1253 Taiwanese females indicated an FI prevalence rate of 2.8% [15]. Studies from China and Korea indicated FI prevalence rates of 1.3% among Chinese women in Beijing and 6.4% among Koreans of both sexes [16,17]. Similarly, studies from the Middle East have reported varying rates of FI prevalence. A 2001 study of 450 females from the United Arab Emirates reported an FI prevalence of 11.3% in [18] while a study of 596 premenopausal Qatari women reported a prevalence rate of 10.4% [19]. To the best of our knowledge, there has been no published information on the prevalence of FI in Malaysia, which is unique in the multi-racial composition of its population.

In order to shed some light on FI among patients in a Malaysian setting, we conducted a survey to determine the prevalence, patient characteristics and risk factors of FI as well as their treatment seeking behaviour. This paper focuses on the first three issues. Details of treatment seeking behaviour will be reported separately.

## Method

A survey was conducted between June 2009 and February 2010 at the University of Malaya Medical Centre (UMMC), an academic hospital in Kuala Lumpur, Malaysia. Based on convenience sampling, survey subjects were recruited among patients seeking treatment at the General Surgical (GS), Obstetrics and Gynaecology (O&G) and Antenatal specialist outpatient clinics as well as their accompanying relatives. Subjects were eligible for inclusion into the study if they were aged more than 18 years old and were of Malaysian nationality. The study had a targeted sample size of 1000 respondents.

Subjects who provided verbal consent to participate in the study were asked to complete a self-administered questionnaire. The questionnaire was specifically designed and pre-tested for this study and included screening questions on demographics, FI symptoms, surgical history and chronic illness. Subjects who reported FI were then asked additional questions regarding the severity of their condition and their treatment-seeking behaviour.

Severity of FI was assessed based on the Wexner Continence Scale (WCS) [20]. The WCS determines severity according to symptoms of incontinence to solid, liquid or gas, wearing of pads and lifestyle alteration. Each WCS domain contains five levels of severity from 0 (never) to 4 (always). The total WCS score ranges from zero (no incontinence) to 20 (complete incontinence). Within the WCS, FI severity is divided into mild (1–4), moderate (5–8) and severe (more than 9) [21].

For the purpose of analysis, numerical variables were transformed into categorical variables and evaluated using percentages. Results were further analysed using the chi-square test with means, medians, standard deviations and inter-quartile ranges (IQR) obtained from descriptive statistical analysis. Analysis was performed using Stata SE version 11.2 (College Station, TX).

Ethical approval for this study was granted by the Medical Ethics Committee of the University Malaya Medical Centre (UMMC) on 20th February 2008 with the document approval number 638.1.

## Results

One thousand subjects comprising of patients and their relatives were recruited to the study. The median age of subjects was 38 years (IQR 24) of whom 76% were female as shown in Table 1. There was a predominance of Malay patients (59.5%), followed by Chinese (20.0%), Indian (17.4%) and patients from other ethnicities (3.1%). Chronic diseases present among 238 (23.8%) of the study subjects included diabetes mellitus (10.8%), hypertension (17.7%), hypercholesterolemia (7.4%) and ischaemic heart disease (2.4%). Two hundred and eighty five (28.5%) of the recruited subjects reported having undergone prior surgery, of whom 1.5% reported having had previous pelvic, perineal or anorectal surgery.

**Table 1:** Sample characteristics

**Characteristic**	**N**	**(%)**
All subjects, N	1000	(100%)
Age, median (IQR)	38	(24)
Female, n(%)	760	(76%)
Ethnicity, n(%)	200	
Chinese	174	(20.0%)
Indian	595	(17.4%)
Malay	31	(59.5%)
Other	83	(3.1%)
Faecal Incontinence	238	(8.3%)
Chronic disease, n(%)	108	(23.8%)
Diabetes mellitus	177	(10.8%)
Hypertension	74	(17.7%)
Hypercholesterolemia	24	(7.4%)
Ischaemic heart disease	285	(2.4%)
Previous operation, n(%)	15	(28.5%)
Pelvic, perineal or anorectal operation	270	(1.5%)
Other operation		(27%)
Defaecation frequency, n(%)		
1–7 times a week	657	(65.7%)
8–15 times a week	244	(24.4%)
16–21 times a week	73	(7.3%)
More than 22 times a week	26	(2.6%)
Defaecation duration, n(%)		
1–5 minutes	547	(54.7%)
6–10 minutes	291	(29.1%)
11–15 minutes	89	(8.9%)
More than 15 minutes	73	(7.3%)

Note: ^Total is 999 as one subject had missing data.

Most of the subjects (65.7%) had a defaecation frequency of 1–7 times per week, while 24.4% and 7.3% of subjects had a defaecation frequency of 8–15 times and 16–21 times, respectively. Twenty six (2.6%) of the subjects reported a frequency of more than 22 times per week. As for the duration of defaecation, 54.7% of subjects reported a defaecation duration of 1–5 minutes, 29.1% reported duration of 6–10 minutes, and 8.9% and 7.3% reported durations of 11–15 minutes and more than 15 minutes respectively as shown in Table 1.

**Figure 1:**
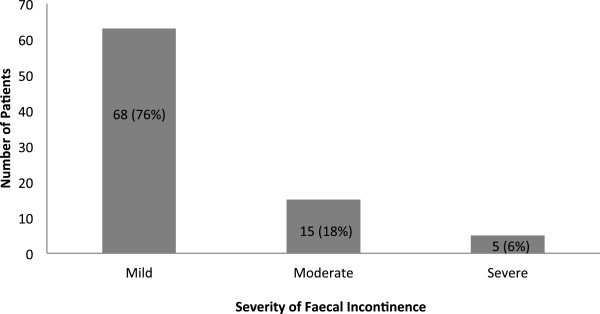
Severity of faecal incontinence as assessed by the Wexner Continence Scale.

Eighty three (8.3%) subjects reported having experienced some form of faecal incontinence as shown in Table 2. The rate of FI was equal between males and females. The median age among the subjects who reported FI was 47 years with an IQR of 28 years. Although the males with FI were older, the age difference between sexes was not statistically significant (52.6 vs. 46.3 years, p = 0.146). Assessed by the WCS, 76% of FI subjects had mild FI (WCS score less than 5) compared to 18% who had moderate FI (WCS score range from 5 to 8) and 6% who had severe FI (WCS score above 8) as shown in Figure 1.

**Table 2:** Relationship between demographic and clinical factors with the presence of faecal incontinence

**Demographic and Clinical factors**	**Survey sample, N**	**Proportion of patients with FI, n (%)**	**Proportion of patient without FI, n (%)**	**p value**
All patients with FI	1000	83(8.3)	917(91.7)	
Age groups, Median (IQR)	38(24)	47(28)	37(24)	<0.001
15–24 years	59	3(5.1)	56(94.9)	
25–44 years	532	32(6.0)	500(94.0)	
45–64 years	318	31(9.7)	287(90.3)	
More than 65 years	91	17(18.7)	74(81.3)	
Sex				0.983
Male	240	20(8.3)	220(91.7)	
Female	760	63(8.3)	697(91.7)	
Ethnicity				0.525
Chinese	200	21(10.5)	179(89.5)	
Indian	595	44(7.4)	551(92.6)	
Malay	174	16(9.2)	158(90.8)	
Other	31	2(6.5)	29(93.5)	
Chronic disease				0.158
Yes	238	25(10.5)	213(89.5)	
No	762	58(7.6)	704(92.4)	
Diabetes mellitus				0.026
Yes	108	15(13.9)	93(86.1)	
No	892	68(7.6)	824(92.4)	
Hypertension				0.323
Yes	177	18(10.2)	159(89.8)	
No	822	65(7.9)	757(92.1)	
Hypercholesterolemia				0.617
Yes	74	5(6.8)	69(93.2)	
No	926	78(8.4)	848(91.6)	
Ischaemic heart disease				0.133
Yes	24	4(16.7%)	20(83.3%)	
No	975	79(8.1%)	896(91.9%)	
Previous operation				0.068
Pelvic, perineal or anorectal operation	141	15(10.6%)	126(89.4%)	
Other operation	144	7(4.9%)	137(95.1%)	
No previous operation	715	61(8.5%)	654(91.5%)	
Defaecation frequency				0.571
1–7 times a week	657	55(8.4%)	602(91.6%)	
8–15 times a week	244	19(7.8%)	225(92.2%)	
16–21 times a week	73	5(6.9%)	68(93.1%)	
More than 22 times a week	26	4(15.4%)	22(84.6%)	
Defaecation duration				0.001
1–5 minutes	547	38(7.0%)	509(93.0%)	
6–10 minutes	291	18(6.2%)	273(93.8%)	
11–15 minutes	89	14(15.7%)	75(84.3%)	
More than 15 minutes	73	13(17.8%)	60(82.2%)	

Note: p value derived from χ2 test.

The prevalence of FI was significantly associated with older age, longer defaecation duration and diabetes mellitus (p < 0.05) as seen in Table 2. FI was found in 18.7% of subjects aged more than 65 years, compared to 9.7% among those aged 45 to 64 years, 6.0% among 24 to 44 year olds and 5.1% among subjects aged 18 to 24 years. The difference in FI rates was highly significant between age groups (p < 0.001). FI was also found to be associated with increased duration of defaecation with a large proportion of patients with FI reporting longer duration of defaecation. FI was significantly associated with the presence of diabetes mellitus (13.9% vs. 7.6%, p = 0.026). No statistical significant relationship was observed between FI and gender (p = 0.983), ethnicity (p = 0.525), weekly defaecation frequency (p = 0.571) or previous operation (p = 0.068).

## Discussion

Our study found that the prevalence of FI was 8.3% among the convenience sample of 1000 patients. This is similar to the findings of some studies and somewhat higher than reported by others as shown in Table 3. The prevalence reported in this study is similar to that reported in Japan [14] and Korea [17] and slightly less than that reported in in UAE [18] or Qatar [19]. There have been, to our knowledge, no previous studies on the prevalence of faecal incontinence in Malaysia. However, another study reported urinary incontinence rate of 9.9% among the elderly in a rural community in Malaysia [22].

**Table 3:** International comparison of the prevalence of faecal incontinence

**Country**	**Author, year**	**Sample description**	**Sample size**	**Age Range (years)**	**Age (years)**						
					**FI and Non-FI**		**FI**		**FI Prevalence (%)**		
					**Mean**	**Median**	**Mean**	**Median**	**Female**	**Male**	**Both**
Japan	Nakanishi, 1997 [14]	Elderly recruited from Osaka	1473	≥65	NA	NA	NA	NA	6.6	8.7	7.65
UAE	Rizk, 2001 [18]	Multiparous females recruited from one medical facility	450	≥20			37.9 ± 13.2		11.3		
Taiwan	Chen, 2003 [15]	Community survey among women in Central Taiwan	1253	≥20	43.2 ± 15.1				2.8		
Qatar	Bener, 2008 [19]	Women visiting primary health care clinics	596	40–48			45.0 ± 0.9		10.4		
China	Ge, 2010 [16]	Women in 6 districts of Beijing	3058	20–79	48 ± 16		63 ± 13		1.28		
Korea	Kang, 2012 [17]	Patients who have undergone medical check‒up from one medical facility	1149	20–82	44.8 ± 10.2		49.0 ± 10.6		6.8	6.2	6.4
Malaysia (present study)	Roslani, 2013	Single site, patients and relatives visiting Obstetrics and Gynaecology and General Surgery Clinics	1000	18-84		38(24)			8.3	8.3	8.3

The wide age range for the selection criteria in our study was similar to several previous studies from UAE, Taiwan, China and Korea (Table 3). The exceptions were a study conducted in Japan in people aged older than 65 years, and another study in Qatar which only included women aged 40 to 48 years old. The median age in our overall study population was 38 (IQR 24) years old. This was slightly younger, albeit with a wide interquartile range, than the means found in other studies. The median age of patients with FI in our study was 47 years old (IQR 28). This is similar to the Korean study by Kang et al. that included male and female patients attending medical check-up at a health facility with a study population age range of 20 to 82 years old, their mean age was 44.8 (±10.2) years old. Kang reported a mean age of 49 (±10.6) years old among FI patients, and an FI prevalence of 6.4%. Other studies in our literature review, focused only on FI among women, reported mean ages of FI patients ranging from 37.9 to 63 years old.

Like several other studies, our study found associations between FI and increasing age, defaecation frequency and diabetes mellitus. Older age has been consistently associated with an increased risk of FI [23]. Kang reported higher prevalence of FI among older age groups (>50 years old) compared to younger groups (<50 years old) with FI prevalence increase gradually with age (10.4% vs. 4.9%, p < 0.001) [17]. Similarly, a previous study from Beijing (China), reported that the mean age of women who had FI was significantly higher compared to the mean age of those without FI (63 vs. 48, p < 0.001) [16].

In our study, FI rates are found to be equal at 8.3% for both males and females. Although one may expect to see a difference in prevalence between male and female patients, our study did not show such an association. The lack of a statistically significant difference in FI rates between genders may have been be due to the small number of positive cases relative to the total sample population. Furthermore, there was a limited number of males, approximately a quarter of the sample size. The US National Health and Nutrition Examination Survey (NHANES) of 2005–2006 found no significant difference in FI between women and men (8.9% vs. 7.7%, p = 0.31) [24]. This was also found in other US studies [2,25], as well as studies for Netherlands [26] and Korea [17]. A study done in Japan among the elderly showed higher prevalence among the women [14].

FI not only occurs in elderly people, but also in patients who have undergone surgery that can affect the excretory organs and related nerves [27,28]. An Australian study reported a significant relationship between FI and episiotomy forceps delivery, perineal tears and hysterectomy in women [28]. In a study of asymptomatic American women, Fox and colleagues reported an association between aging and reduced anorectal function and suggested that aging plays a part in declining anorectal function and may worsen effects of any earlier damage [29]. The present study did not find surgical history to be a significant risk factor although this could be due to inadequate surgical history obtained from our subjects.

Constipation is also one of the significant risk factors related to FI. A previous study among Norwegian women showed that the prevalence of FI increased in women who have chronic constipation compared to those who did not (3.8%, 95% CI 2.8-4.7 vs. 2.9%, 95% CI 2.7-3.2) [30]. Similarly, in a recent study conducted in the USA, the prevalence of FI was higher in constipated compared to non-constipated women (14% vs. 8%, p < 0.001) while constipation was also more prevalent among incontinent compared to continent women (43% vs. 30%, p < 0.001) [31]. Our results are consistent with previous findings, showing longer duration of defaecation associated with FI. Longer duration of defaecation indicates constipation, as patients have to spend more time on the toilet.

FI has also been shown to be related to chronic illness, mainly inflammatory bowel disease, irritable bowel syndrome, multiple sclerosis and diabetes mellitus [32]. In this study, we found FI was associated with diabetes mellitus (13.9% vs. 7.6%, p = 0.026). Other studies have also shown FI to be a concern for diabetics due to damage to the nervous system caused by long-standing diabetes [33-35]. A majority of patients in this study had mild incontinence (76%) and only 6.0% had severe incontinence as measured by the WCS. We did not find any association between risk factors and the severity of FI.

There are several limitations in the current study. Subjects in the study were drawn from a convenience sample of patients and their accompanying relatives recruited from the O&G, General Surgery and Antenatal clinics of the UMMC. As an initial exploratory study, we did not recruit respondents from other specialist clinics in UMMC. However, we recognize that the sampling method and location limits its generalizability to the larger Malaysian population. Although the results cannot be considered to be representative of the entire Malaysian population, it may give some indication of occurrence of the condition in the absence of a more representative study. Furthermore, the recruitment of respondents from the antenatal and O&G clinics may give rise to concern about a preponderance of women with obstetric complications leading to FI. However, the prevalence of FI in our study sample is similar to other reported studies. Lastly, FI in this study was self-reported by subjects based on their answers to the questionnaire and their understanding of what was asked. The self-reporting format may allow for more anonymity to the patient, so we think that the results provide a reasonable indication of the disease prevalence in patients at this hospital setting. Despite these limitations, we believe the findings are worth considering for various reasons. Among them are that the study recruited a large sample of one thousand subjects and included not only patients, but people who accompanied them to the clinic.

## Conclusions

To the best of our knowledge, this is the first study to estimate the prevalence of FI in a population of Malaysian patients as well as estimate its severity and the risk factors associated with the condition. The results of our study suggest that FI in our setting is prevalent enough to warrant targeted healthcare interventions, including the need to improve general public awareness of the condition, in order to counter social stigma and embarrassment that may be faced by patients. Further study, preferably in a more representative sample of the population, ought to be conducted to determine the extent of FI among Malaysians.

## Competing interests

The authors declare that they have no competing interests.

## Author contributions

ACR designed the study, and participated in the data collection, analysis and drafting the manuscript. RR drafted the manuscript. RR was involved in data collection and analysis. SA participated in the analysis and drafting of the manuscript. DJA and AG were involved in analysis. All authors read and approved the final manuscript.

## Pre-publication history

The pre-publication history for this paper can be accessed here:

http://www.biomedcentral.com/1471-230X/14/95/prepub
